# Local inhibition of rRNA transcription without nucleolar segregation after targeted ion irradiation of the nucleolus

**DOI:** 10.1242/jcs.232181

**Published:** 2019-10-01

**Authors:** Christian Siebenwirth, Christoph Greubel, Guido A. Drexler, Judith Reindl, Dietrich W. M. Walsh, Benjamin Schwarz, Matthias Sammer, Iris Baur, Helmut Pospiech, Thomas E. Schmid, Günther Dollinger, Anna A. Friedl

**Affiliations:** 1 Bundeswehr Institute of Radiobiology, 80937 Munich, Germany; 2 Institute for Applied Physics and Metrology, Universität der Bundeswehr München, 85577 Neubiberg, Germany; 3 Department of Radiation Therapy and Radiooncology, Technical University of Munich, 81675 Munich, Germany; 4 Department of Radiation Oncology, University Hospital, Ludwig Maximilians University of Munich, 81377 Munich, Germany; 5 Leibniz Institute for Age Research – Fritz Lipmann Institute (FLI), 07745 Jena, Germany

**Keywords:** DSB, Ion microbeam, Nucleolus, rRNA transcription

## Abstract

Nucleoli have attracted interest for their role as cellular stress sensors and as potential targets for cancer treatment. The effect of DNA double-strand breaks (DSBs) in nucleoli on rRNA transcription and nucleolar organisation appears to depend on the agent used to introduce DSBs, DSB frequency and the presence (or not) of DSBs outside the nucleoli. To address the controversy, we targeted nucleoli with carbon ions at the ion microbeam SNAKE. Localized ion irradiation with 1–100 carbon ions per point (about 0.3–30 Gy per nucleus) did not lead to overall reduced ribonucleotide incorporation in the targeted nucleolus or other nucleoli of the same cell. However, both 5-ethynyluridine incorporation and Parp1 protein levels were locally decreased at the damaged nucleolar chromatin regions marked by γH2AX, suggesting localized inhibition of rRNA transcription. This locally restricted transcriptional inhibition was not accompanied by nucleolar segregation, a structural reorganisation observed after inhibition of rRNA transcription by treatment with actinomycin D or UV irradiation. The presented data indicate that even multiple complex DSBs do not lead to a pan-nucleolar response if they affect only a subnucleolar region.

## Abstract (precis)

**Summary:** Highly localized damage induced in nucleoli by carbon ion irradiation at the microbeam SNAKE only locally inhibits rRNA transcription, but does not cause nucleolar segregation.

## INTRODUCTION

Nucleoli are the largest organelles within the cell nucleus. In addition to their function in transcription and processing of ribosomal RNA (rRNA), they have attracted interest for their role as cellular stress sensors and as potential therapeutic targets for cancer treatment (Boulon et al., 2010; Hein et al., 2013; Lindström et al., 2018). Nucleoli harbour several hundred copies of 13 kb ribosomal DNA (rDNA) coding sequences, each separated by intergenic sequences (IGS) of about 30 kb length. The transcriptionally competent coding regions seem largely devoid of nucleosomes, but they are covered with the architectural transcription factor UBF, whereas IGS regions exhibit a nucleosomal structure (O'Sullivan et al., 2002; Zentner et al., 2011; Herdman et al., 2017). Cells expressing fluorescence-tagged histones show fibres of intranucleolar condensed chromatin (ICC) in close contact with fibrillary centres (FCs) (Tchelidze et al., 2017). The ICC fibres seem to connect with perinucleolar condensed chromatin (PCC), which surrounds the nucleoli.

Under conditions allowing RNA polymerase I (RNA Pol I)-dependent transcription, nucleoli exhibit a tripartite structure, with a number of FCs surrounded by dense fibrillary component (DFC) in each nucleolus (reviewed by Mangan et al., 2017; Schöfer and Weipoltshammer, 2018). rDNA is located within the FCs, and UBF is generally used as a marker of FC domains. Transcription of 45S pre-ribosomal RNA (pre-rRNA) takes place at the interface of FCs and DFCs. Nascent pre-rRNA is immediately transported to DFC zones, where early processing of pre-rRNA takes place. Late processing steps take place in the granular component, which makes up the remainder of the nucleoli and in which the FCs and DFCs are embedded.

In response to inhibition of rDNA transcription (e.g. by treatment with actinomycin D), numerous small (0.3–0.5 µm diameter) UBF-positive foci coalesce into a few larger intensive UBF foci, which finally form a few crescent-like caps associated with prominent DFC zones at the rim of the nucleoli. The whole process is accompanied by a reshaping that makes the nucleoli more spherical than under transcription activity. This nucleolar segregation has also been observed after treatment of cells with a variety of stressors, such as UV irradiatoin, heat shock and overexpression of TOPBP1 (Al-Baker et al., 2004; Boulon et al., 2010; Moore et al., 2011; Sokka et al., 2015). In general, nucleolar segregation is associated with silencing of rDNA transcription, as demonstrated by a drastic reduction in ribonucleotide incorporation at nucleolar regions. It is generally assumed that cessation of transcription and nucleolar segregation are causally linked.

In recent years, the effect of induction of DSBs on transcription of genes carrying the DSB or adjacent genes has attracted interest. The data obtained are not without contradictions, but the following picture emerges for RNA polymerase II-transcribed genes: Transcription of genes carrying a DSB is reduced, as shown by transcript analyses after site-specific enzymatic induction of breaks (Iacovoni et al., 2010; Shanbhag et al., 2010; Pankotai et al., 2012; Iannelli et al., 2017; Abu-Zhayia et al., 2018). This reduction is mediated by DNA damage repair (DDR) kinases [e.g. ATM and the DNA-PK complex) and is not just a passive effect of disturbance of RNA polymerase II by the presence of the break. Several, but not all, analyses also observed a reduction in transcription or accumulation of repressive chromatin marks for genes at a distance up to several 100 kb from the site-specific break site (Ayrapetov et al., 2014; Gong et al., 2017; Iannelli et al., 2017). Similarly, we and several others have observed exclusion of ribonucleotide incorporation or of RNA polymerase II elongation in regions marked by γH2AX that surround DSB sites induced by ionizing radiation, laser irradiation or enzyme action (Seiler et al., 2011; Penterling et al., 2016; Gong et al., 2017). γH2AX domains have an estimated size of 1–2 Mbp; thus, these data correlate roughly with the extent of transcriptional suppression adjacent to the damage site.

For transcription in nucleoli, the emerging picture seems less complete, although one of the first investigations of the effect of break induction on local transcription was performed in nucleoli (Kruhlak et al., 2007). After ionizing irradiation, which leads to random distribution of hits in nucleoplasm and nucleoli, a silencing of RNA Pol I-mediated transcription, mostly inferred from reduced ribonucleotide incorporation, was described by several authors (Kruhlak et al., 2007; Calkins et al., 2013; Larsen et al., 2014), whereas others did not observe reduced transcription (Moore et al., 2011; Warmerdam et al., 2016). Reduced ribonucleotide incorporation was also observed after nucleolar induction of DSBs by means of homing endonuclease I-PpoI or CRISPR/Cas9 (Harding et al., 2015; Van Sluis and McStay, 2015; Warmerdam et al., 2016; Pefani et al., 2018), methods that induce a very high number of breaks in all rDNA regions. It has been suggested that the transcriptional response differs when damage induction is confined to one or a few nucleoli compared with situations where the nucleoplasm is also hit (Larsen and Stucki, 2016). The latter scenario was proposed to induce a response *in trans* in all nucleoli of the cell, even in nucleoli that were not hit by irradiation (Kruhlak et al., 2007; Larsen et al., 2014), whereas response to the former was confined to nucleoli that were actually hit (Kruhlak et al., 2007). To our knowledge, the study by Kruhlak et al. (2007) is the only one in which DSBs were induced specifically in one nucleolus so that the response of the other nucleoli could be studied. These authors used laser microirradiation and painted the whole targeted nucleolus with irradiation, which led to reduced transcription in the whole nucleolus. Several issues arise: (1) The number of DSBs induced by laser microirradiation is largely unknown and estimates rely on the study of response reactions such as H2AX phosphorylation, rather than the breaks themselves, thus making comparisons difficult. (2) Because the whole nucleolus was targeted, the spatial extension of the response to single DSBs could not be evaluated. (3) It cannot be excluded that laser microirradiation in addition to DSBs induces so-called bulky lesions, which are known to elicit a specific response by stalling RNA polymerase (Wickramasinghe and Venkitaraman, 2016), in contrast to base damage associated with ionizing radiation, which is not bulky.

Nucleolar segregation was observed after ionizing irradiation by some authors (Kruhlak et al., 2007), but not by others (Moore et al., 2011; Foltánková et al., 2013). After DSB induction by I-PpoI or CRISPR/Cas9, nucleolar segregation appears to be generally seen (Harding et al., 2015; Van Sluis and McStay, 2015; Franek et al., 2016; Pefani et al., 2018). It has been suggested that nucleolar reorganisation only occurs under conditions with very high numbers of DSBs (Larsen and Stucki, 2016). To our knowledge, the effect of targeted DSB induction in single nucleoli on nucleolar segregation of targeted and non-targeted nucleoli has not yet been investigated.

To further elucidate the response to DSB induction in single nucleoli, we established targeted irradiation of single nucleoli with defined numbers of carbon ions (initial energy 55 MeV and 43 MeV at the cell layer) (Siebenwirth et al., 2015), for which well-characterized estimates of the number of DSBs induced are available (Hauptner et al., 2006). To study the effect of higher damage load, the local dose in the nucleoli was varied by varying the number of ions delivered to a submicrometre spot. We observed no global reduction in ribonucleotide incorporation in the targeted nucleolus or in other nucleoli of the same cell. However, both 5-ethynyluridine (5EU) incorporation and poly [ADP-ribose] polymerase 1 (Parp1) protein levels were locally decreased at the damaged nucleolar chromatin regions marked by γH2AX, suggesting localized inhibition of rRNA transcription. This locally restricted transcriptional inhibition was not accompanied by nucleolar segregation. We conclude that ionizing radiation, even at high doses, induces only localized repression of rDNA transcription, but no nucleolar segregation or other global responses.

## RESULTS

### Ion microirradiation allows targeted damage induction

**Fig. 1.**
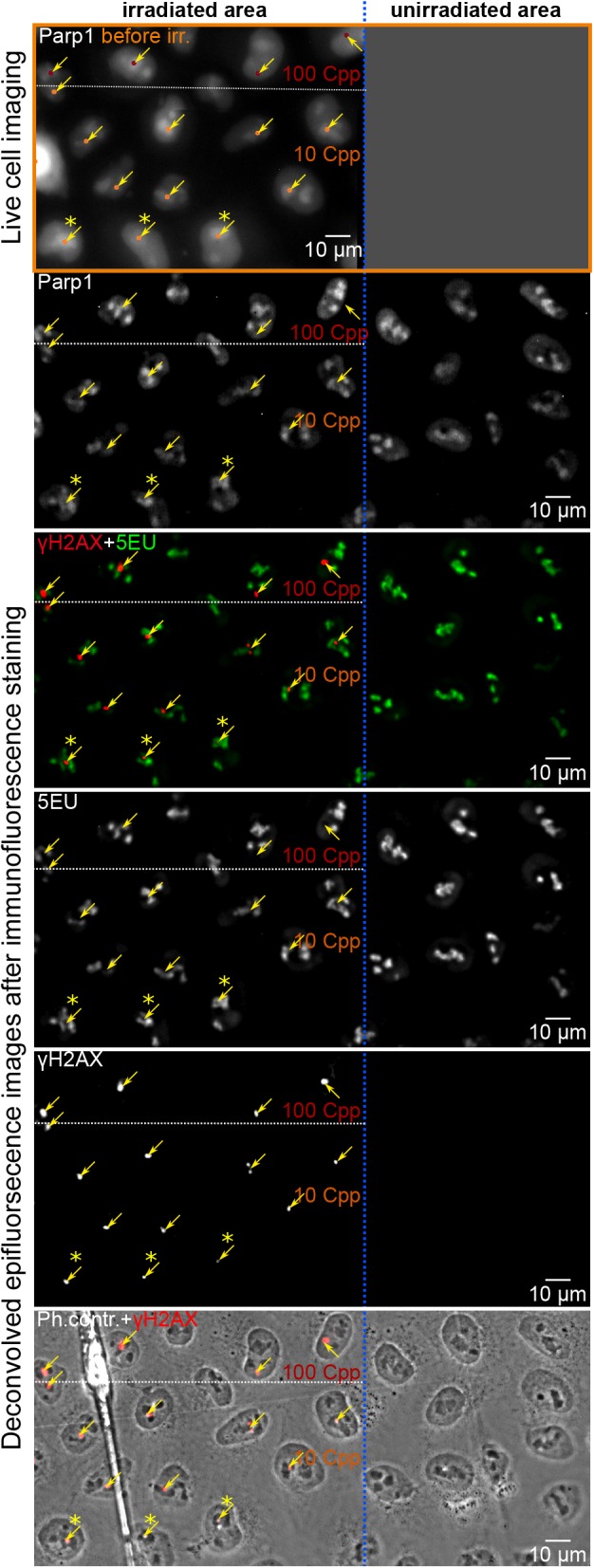
**Area of irradiated and unirradiated HeLa-Parp1**–**CB-tagRFP cells.** Cells on the left side of the dotted blue line were irradiated with 10 or 100 Cpp (orange and red target points, respectively) and allowed to recover for 1.5 h. For targeting, a single slice image of the Parp1 signal of the cells was taken (orange frame). Neighbouring cells on the right side of the blue dotted line were not irradiated and were used as controls. After 5EU incorporation and immunofluorescence staining, cells were imaged as *z*-stacks of 33 layers, with 300 nm distance in the single channels of Parp1, 5EU, γH2AX and phase contrast. Stacks were deconvolved for improved depth resolution. A single slice from the centre of the stack is shown. Arrows mark the target and hit areas verified by γH2AX. The best examples are the hit nucleoli in the three bottom cells (marked by asterisks). The outlines of these nucleoli in the phase contrast image still correlate with the outline before irradiation. The γH2AX foci are completely embedded in the nucleoli and at their sites; 5EU and Parp1 signals are less intense as in direct proximity. However, the irradiated nucleoli and the residual nucleoli of the corresponding nucleus do not show an overall decrease in 5EU signal. Fluctuations in brightness correlate with Parp1 fluctuations before irradiation and are also observable in control cells.

Parp1 accumulation in nucleoli is a well-characterized feature (Yung et al., 2004; Rancourt and Satoh, 2009). As expected, HeLa Parp1–CB-tagRFP cells expressing an RFP-tagged chromobody (CB) that binds Parp1 (Buchfellner et al., 2016) demonstrate nucleolar accumulation of endogenous Parp1 protein (Fig. 1), a fact that we exploited for targeted irradiation of nucleoli. Nucleoli of more than 1500 cells were irradiated with 1, 10, 50 or 100 carbon ions per point (Cpp). The beam spot size was less than 1 µm full-width at half maximum (FWHM) after targeting several ions to one spot. In analogy with the calculations of Hauptner et al., 2006, for single 43 MeV carbon ions 50% of the dose was concentrated in a radius of less than 10 nm around the ion trajectory and nearly all ionizations were within a radius of 500 nm. Irradiation was targeted to the centre of one nucleolus per cell (typically the largest one) during the target definition step, which took place 30–120 s before the actual irradiation The recovery times after irradiation were 15 min, 45 min, 1 h 15 min, 1 h 30 min, 1 h 45 min, 2 h 15 min, 3 h 15 min up to 6 h, 6 h 30 min and 7 h. After the recovery time, ribonucleotide incorporation was monitored by 30 min of pulse labelling with 5EU. Immediately afterwards, cells were fixed and subjected to immunofluorescence (IF) detection of γH2AX and visualization of 5EU. Every single cell was imaged for tagRFP (Parp1), Alexa Fluor 488 (5EU) and Alexa Fluor 647 (γH2AX) signals as well as in phase contrast.

Fig. 1 shows typical examples of pre-irradiation Parp1 signals and the intended target sites (orange and red dots for irradiation with 10 and 100 Cpp, respectively), together with the registered nuclei after IF detection of γH2AX and 5EU incorporation. All γH2AX foci were categorized according to form (single, double or multiple foci and banana foci; see Fig. S1A) and location in relation to the nucleolar 5EU signal (see Fig. S1B). In about 40% of irradiated nucleoli, a single γH2AX focus was seen after irradiation and, due to its large variance, this proportion appeared to be independent of irradiation dose and recovery time (pink triangles, Fig. S1C; green triangles, Fig. S2A). As expected, the size of the single foci tended to become larger with increasing number of carbon ions delivered at the sites (Fig. S1D).

In the next step, nucleoli in which the γH2AX signal was surrounded in *x*- and *y*-directions by Parp1 signal were evaluated further, as these nucleoli were considered to be certain nucleolus hits. Their proportion increased with the number of ions per point from about 30–40% of all evaluated foci (black squares, Fig. S1E), which might result from a higher hitting probability with increasing ion number. The frequency of certainly missed nucleoli was below 10% at all doses (pink triangles, Fig. S1E), indicating the high accuracy of the targeted irradiation procedure.

The yields of certain hits showed large variance, but did not show a time-dependent trend (green triangles; Fig. S2B). These analyses indicate that there is no directed γH2AX foci movement to a location at the periphery or outside the nucleoli during post-damage processing

In summary, nucleoli with a single γH2AX focus are by far the most common outcome of targeted nucleolar irradiation with carbon ions (Fig. S1C). To evaluate the effects of the nucleolar DNA damage, only single foci considered as certain hits (surrounded by Parp1 and 5EU signal) were studied further. As seen in Fig. S1F, their frequency (black squares) was about 10–20% of all irradiated nucleoli. In total, this corresponded to about 160 γH2AX foci that could be included in further analyses.

### Targeted irradiation of nucleoli results in localized loss of 5EU incorporation confined to γH2AX-decorated regions

An overlay of γH2AX and 5EU signals demonstrated that incorporation of 5EU was not stopped in all irradiated nucleoli (Fig. 1). Overall, 5EU signal intensities of targeted nucleoli were comparable to those of untargeted nucleoli in the same cells. We did not find any indication of pan-nuclear reduction in 5EU incorporation (compare irradiated and unirradiated cells in Fig. 1), although it was expected that some DNA damage would be induced in the nucleoplasm lying in the track direction underneath and above the nucleoli (see Discussion). Even a broader spatial distribution of radiation dose, as obtained by irradiating with cross patterns, did not induce a pan-nuclear effect on rRNA transcription (see Fig. S3).

**Fig. 2.**
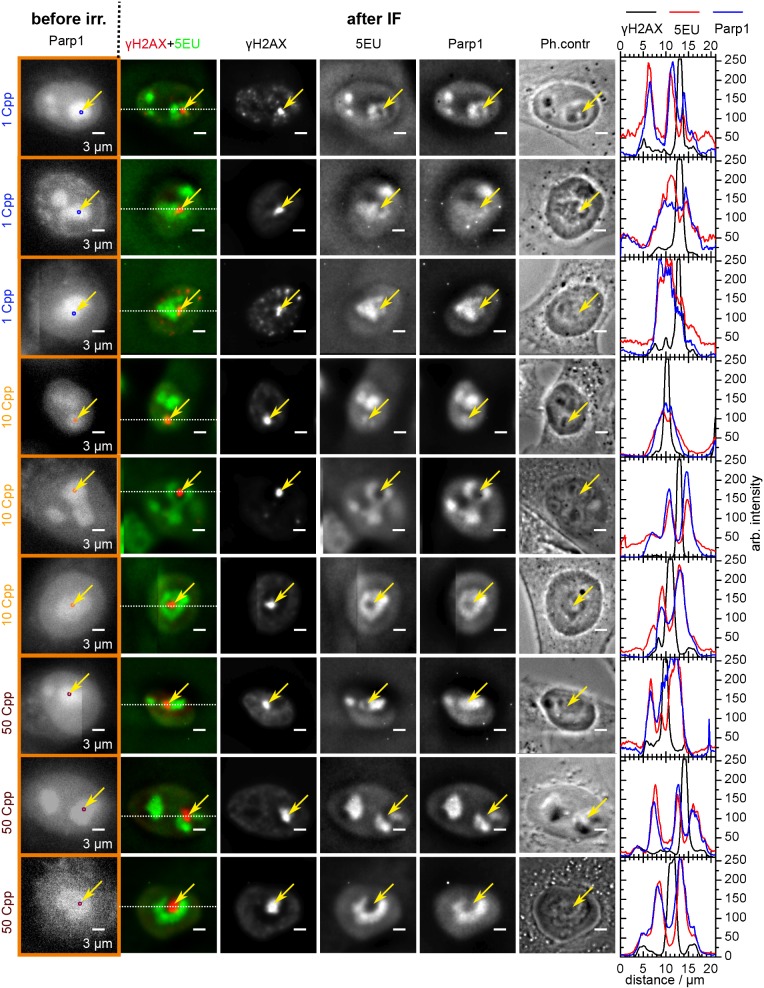
**Certain nucleoli hits after irradiation with 1, 10 and 50 Cpp and their influence on rRNA transcription.** Recovery time was between 30 min and 7 h. Target definition images before irradiation are shown on the left. γH2AX, 5EU, Parp1 and phase contrast images of single cells were taken in the focus depth of the γH2AX foci after IF staining. Additionally, intensity line plots through the γH2AX foci along the white dotted line of the merged γH2AX+5EU image are shown on the right. Contrast was chosen such that 0.1% of the pixels were in saturation. The intensity plots show how 5EU and Parp1 signals (red and blue, respectively) were locally reduced at the damage site marked by γH2AX (black). Two-dimensional location is marked by yellow arrows. In these images, this correlates with local reduction of Parp1 and at higher doses additionally with altered appearance of the nucleolus in the phase contrast image. For better cell reconstruction, we used tiled images that consisted of 2×2 fields of view from the camera before irradiation and 3×3 after IF staining; thus, some cell images show the edge of the tiles. Scale bars: 3 µm.

**Fig. 3.**
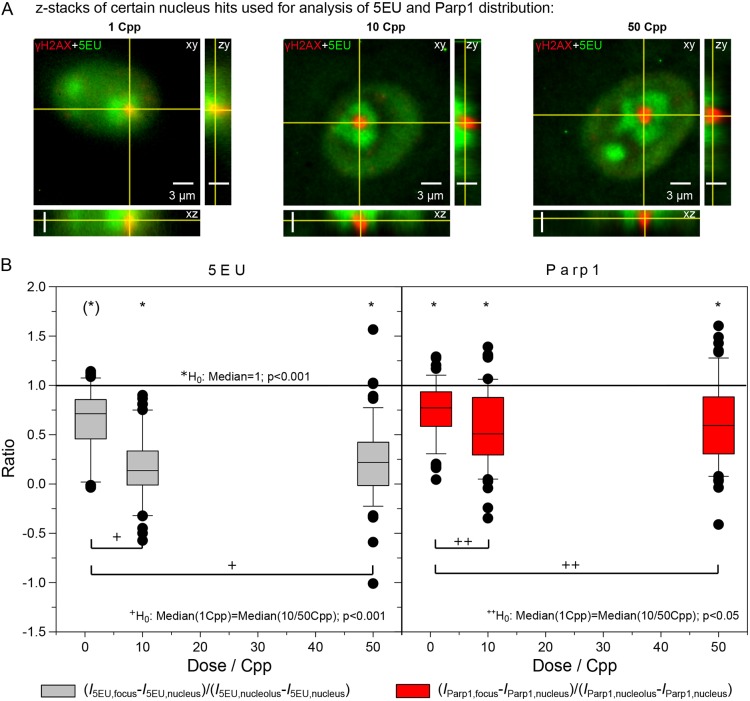
**Cross-sections through *z*-stacks of certain nucleolus hit examples used for 5EU and Parp1 analysis.** (A) 5EU and Parp1 signals were analysed in the central slice of the γH2AX foci (compare with Fig. 2). Nucleolar signal of 5EU is in green and γH2AX in red. The *z*-stacks shown consist of 13 slices with distance 300 nm, containing information of the single nucleus. (B) Analysis of single foci showing certain nucleolus hits on 5EU incorporation (left) and Parp1 distribution (right). Boxplots show median of the 5EU and Parp1 intensity (*I*_5EU_, *I*_Parp1_) ratios, in which the box represents the 2nd quartile, error bars show 1.5× the interquartile range and dots indicate outliers of the distribution. The signal intensity at the focus *I*_…,focus_ minus the nucleus background signal *I*_…,nucleus_ was calculated as a ratio of the signal intensity in the residual nucleolus *I*_…,nucleolus_ minus the nucleus background signal *I*_…,nucleus_. 5EU and Parp1 signals were significantly reduced at the DNA damage site marked by γH2AX. A one-sample signed rank test (*) was used to test the hypothesis H_0_: median=1, which gave *P*<0.001. For the higher rates of 10 and 50 Cpp, 5EU incorporation and Parp1 intensity significantly decreased to about 0.2 and 0.5, respectively. The Mann–Whitney rank sum test was used to test the difference of the medians for 10 or 50 Cpp compared with 1 Cpp, which gave ^+^*P*<0.001 for 5EU and ^++^*P*<0.05 for Parp1. Scale bars: 3 µm (for all cross-sections).

Importantly, at close inspection it was evident that both 5EU incorporation and the Parp1 signal were locally decreased at the damage site marked by γH2AX (images showing typical γH2AX foci localizations with regard to 5EU and Parp1 are shown in Fig. 2 and examples of corresponding cross-sections of z-stacks in Fig. 3A). Note the excellent agreement between 5EU and Parp1 signals, and the localized loss of signals at the position of the γH2AX foci. This was further elucidated in the intensity plots along the white dotted line through the γH2AX foci (Fig. 2). Thus, after targeted ion irradiation, transcriptional silencing appears to be restricted to a volume very close to the ion track, which, within the limits of microscopic resolution, coincides with the volume decorated by γH2AX.

**Fig. 4.**
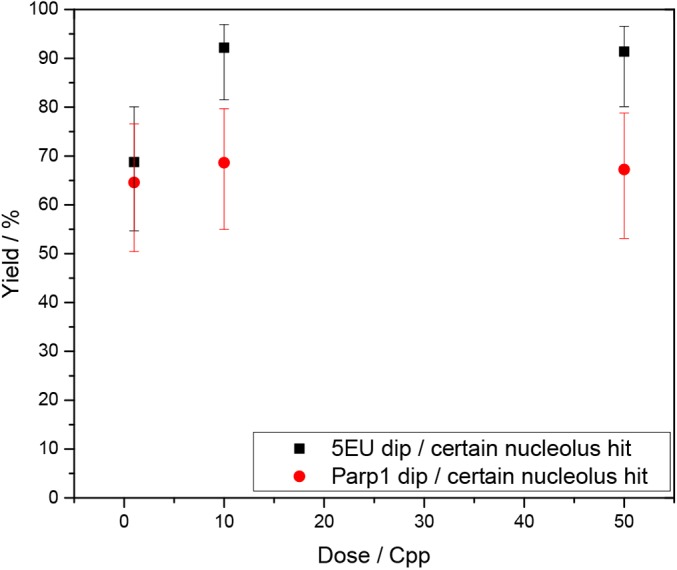
**Analysis of single foci showing certain nucleolus hits on 5EU incorporation and Parp1 distribution.** Frequency of single foci, showing certain nucleolus hits with dips in the 5EU and Parp1 signals. If the ratio was below 0.80, the area was defined as a dip. Error bars describe the 95% confidence Wilson score intervals of the binomial proportion.

The average reduction in 5EU signal intensity in the ‘dips’ was determined. After irradiation with one ion (corresponding to 0.3 Gy in the nucleus), the median of the 5EU intensities at a γH2AX focus (corrected for the 5EU background in the nucleus) was significantly reduced to 0.64, compared with the residual 5EU signal of the affected nucleolus (corrected for the 5EU nucleus background). After 10 and 50 ions per point, the intensity ratio for 5EU dropped significantly further to 0.14 and 0.22, respectively (Fig. 3B; left). The localized reduction in 5EU incorporation was paralleled by a decrease in Parp1 intensity, which was less pronounced, but still significant. For the null hypothesis H_0_: median(1,10, 50 Cpp)=1, *P*<0.001; for H_0:_ median(1 Cpp)=median(10 or 50 Cpp), *P*<0.001 for 5EU and *P*<0.05 for Parp1 (Fig. 3). Statistical analysis was performed using the Mann–Whitney rank test. After irradiation with at least 10 carbon ions per point, more than 90% of the certain nucleolar γH2AX foci showed a 5EU dip of <0.80 (Fig. 4). No significant changes were noticed in these phenomena over the studied recovery times (15 min to 7 h) (Fig. S4A,B,C).

### Targeted ion irradiation of nucleoli does not result in relocalisation of UBF

**Fig. 5.**
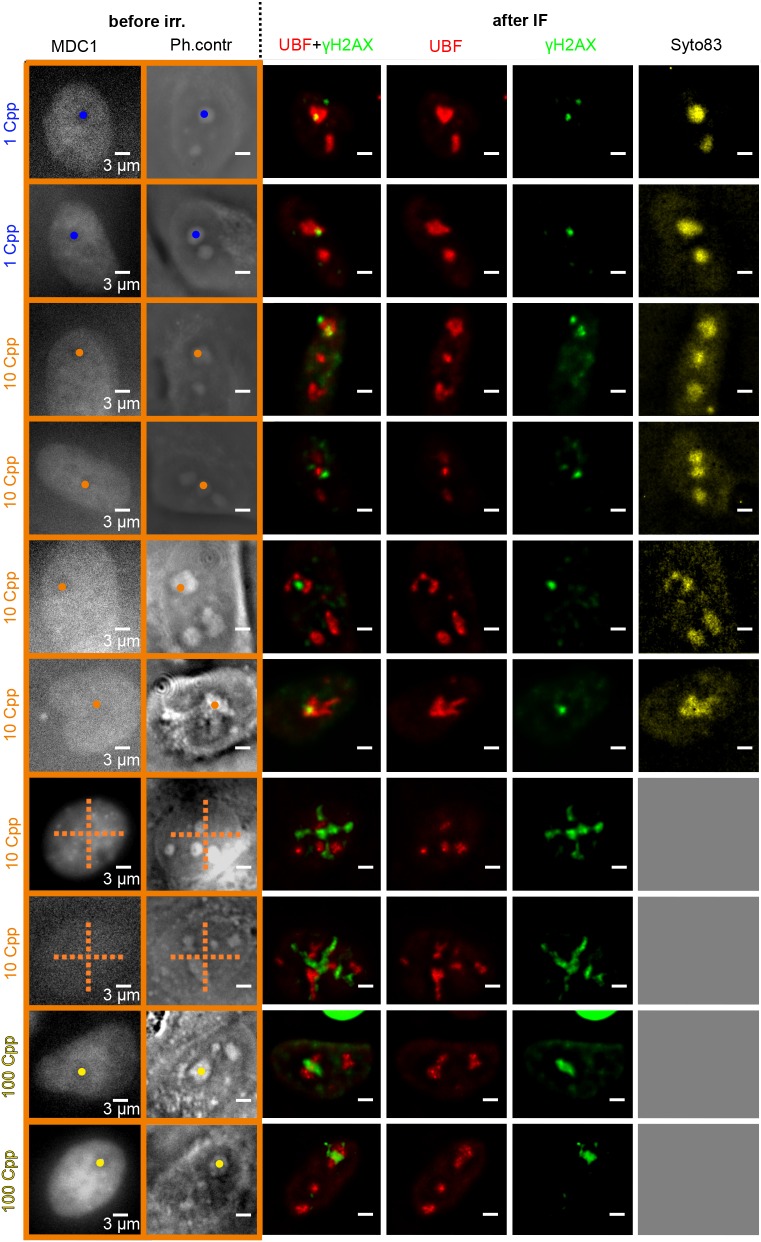
**Effect of targeted irradiation with 1, 10 and 100 carbon ions on the distribution of UBF in stably transfected MDC1-GFP-U2OS cells.** The last two rows of the 10 Cpp irradiations show cross irradiations, in which 170 ions were distributed in the nucleus. For targeting, phase contrast and MDC1–GFP live-cell images were used (orange outline). After IF staining of UBF and damage by γH2AX 2–3 h after irradiation, UBF showed its typical speckled appearance. Staining of RNA by Syto83 indicated that UBF speckles were distributed within the remaining nucleoli area and did not form the typical caps in the periphery of segregated nucleoli. Cap-like UBF signals were not seen in irradiated or unirradiated nucleoli at any dose. For 10 and 100 Cpp, targeted nucleoli partly changed their shape compared with before irradiation and were bent around the γH2AX foci or even showed a displaced appearance around the foci (fifth row). These changes were not the typical segregation characterized by formation of smaller spherical nucleoli accompanied by UBF caps. Thus, we conclude that our ion irradiation does not cause nucleolar segregation. Scale bars: 3 µm.

Nucleolar segregation marked by relocalisation of UBF domains into nucleolar cap structures after treatments that induce pan-nucleolar blockage of rDNA transcription and nucleolar segregation, such as treatment with actinomycin D, is well described (Al-Baker et al., 2004; Boulon et al., 2010; Moore et al., 2011; Sokka et al., 2015). We observed distribution of UBF and fibrillarin in cap-like structures at the periphery of spherical nucleolar regions stainable by NOP52 (marker of the granular component) and Syto83 (RNA marker) in response to actinomycin D treatment that was clearly differentiable from distribution during the transcription-competent state (Fig. S5A,B,C). To investigate whether localized reduction in 5EU incorporation after targeted irradiation is associated with UBF redistribution, we used U2OS cells stably transfected to express DNA damage response factor Mdc1 fused with GFP. Using live-cell imaging, the localization of nucleoli was evident from reduced GFP signals (Fig. 5). For better visualization of the irradiation damage, we additionally stained γH2AX with Alexa Fluor 488 in the same colour channel as the DNA repair protein Mdc1. After irradiation, no major disturbance of UBF distribution was observable and UBF speckles were distributed over the whole nucleolar region, as stained by Syto83. In no instance did we see the typical caps at the periphery of segregated nucleoli. In addition, targeted nucleoli kept their elongated or irregular form and lacked the spherical appearance that is typical for nucleoli undergoing segregation (Tchelidze et al., 2017). After irradiation with 10 Cpp and 100 Cpp in targeted nucleoli, we observed localized reduction of UBF signal intensity at γH2AX sites. In rare cases, this led to an appearance similar to crescent-like UBF caps (Fig. 5, row 5), but the remaining UBF signal still coincided with the Syto83 signal rather than concentrating at its periphery. Note that nucleoli did not adopt spherical morphology, even at very high doses (e.g. Fig. 5, rows 9 and 10). Thus, we concluded that targeted ion irradiation does not cause segregation of the targeted nucleolus, even at an estimated nucleus dose of 30 Gy delivered to a single nucleolus irradiated with 100 Cpp. Furthermore, irradiation using a cross pattern, where the nucleoplasm is also hit with a high dose (corresponding to ∼50 Gy), did not induce pan-nucleolar segregation.

## DISCUSSION

To date, targeted DNA damage in the nucleolar region has been generated by UVA laser microirradiation in presensitized cells (Kruhlak et al., 2007; Larsen et al., 2014) or by endonucleases (Harding et al., 2015; Van Sluis and McStay, 2015; Franek et al., 2016; Warmerdam et al., 2016; Pefani et al., 2018). UVA laser irradiation enables the targeted induction of damage at a certain area and time and, thus, the targeted observation of its effect. On the other hand, the applied energy in the target area is hard to determine, making it difficult to compare results from different laboratories. In addition to DSBs, UVA irradiation in presensitized cells is known to induce cyclobutane pyrimidine dimers (CPDs) and possibly 6–4 photoproducts (Dinant et al., 2007; Kong et al., 2009). These lesions can stall transcription and replication (Tommasi et al., 1996; Selby et al., 1997; Mei Kwei et al., 2004; Edenberg et al., 2014) and thus can strongly affect the interpretation of results. Indeed, it has been shown that these primary UV-induced damages are very efficient at blocking rDNA transcription and/or inducing nucleolar segregation (Al-Baker et al., 2004; Chen et al., 2018).

Inducible expression of endonucleases seems to be a good tool for specifically inducing DSBs in the rDNA and the homing endonuclease I-PpoI, which recognizes a target sequence in 28S rDNA, has frequently been used. Prolonged presence of the endonuclease results in repeated cutting and rejoining cycles until misrepair alters the target sequence. It has been estimated that about 20% of the identified target sites are cleaved, which would generate about 60 DSBs per cell (Van Sluis and McStay, 2015), with all nucleoli affected by DSBs. It should be noted that some additional target sites are present outside the rDNA (Muscarella et al., 1990).

For the first time, this work presents targeted ion microbeam irradiation as a method that is able to apply a defined number of ions, and thus well-defined ionizing radiation damage, in a selected nucleolus at a specified time. Ions with high linear energy transfer (LET), such as the carbon ions used here, directly cause DSBs, single-strand breaks, oxidized bases and apurinic-apyrimidinic sites (Hada and Georgakilas, 2008). Using targeted irradiation, it is possible to correlate the time of irradiation with the effect. This method represents a powerful tool for understanding the effects of damage induced by ionizing irradiation on the nucleolus and carries the potential to translate observations obtained (e.g. by using endonucleases) into radiation research and potentially radiotherapy.

For the presented experiments, we used 55 MeV carbon ions, which have a LET value of approximately 360 keV/µm at the cell layer. With the dimensions of the target volume (density ρ, height *h* and area *A*) known, a specific number (*N*) of carbon ions in that volume deposits a dose *D*, where *D*=*N* *LET* *h/(ρ* *A* *h*), allowing estimation of the DNA damage caused. Assuming a height of 6 µm, an area of 170 µm^2^ and a density of water of 1 g/cm^3^, the dose of one carbon ion in the nucleus of human cells such as HeLa or U2OS cells is approximately 0.3 Gy. Based on about 30 DSBs/Gy induced by low LET radiation, this dose would result in an estimated number of 1.5 DSBs/µm ion track in the nucleus, which could be seen as a lower estimate for high LET particles. In contrast to sparsely ionizing X-ray radiation, used carbon ions induce clustered ionizations that mainly concentrate in less than 100 nm around the ion track. Assuming an enhanced relative biological effectiveness of 2.5 for DSB induction by 55 MeV carbon ions compared with sparsely ionizing radiation (Hauptner et al., 2006), about 4 DSBs/µm track length are expected in nuclear DNA.

For DNA in nucleoli, this estimate has to be adjusted. In human cells such as HeLa and U2OS, 0.4% of the genome represents coding rDNA and about half of all nucleolar organiser regions appear to be silent and not forming nucleoli (McStay and Grummt, 2008; Schöfer and Weipoltshammer, 2018). According to three-dimensional (3D) imaging of HeLa cells stained for H3K9me3 (Fig. S5C) (Tchelidze et al., 2017), approximately 5% of the nucleus volume is occupied by rDNA-containing nucleoli. This leads to the estimate that the nucleolar DNA density is about 5% of the average DNA density of the whole nucleus and, therefore, one single carbon ion should cause 0.2 DSBs/µm in the nucleolus. Variation in the number of ions at the target from 1 to 100 is expected to vary the damage from 0.2 to 20 DSBs/µm in a hit nucleolus and from 4 to 400 DSBs/µm in the surrounding nucleus.

The above-mentioned 3D observations also show an average nucleolus height of approximately 50% of the nucleus height. Thus, at least several DSBs are expected in the nuclear DNA above and beneath the targeted nucleolus, which guarantees reconstruction of the hit position even if damage in the nucleolus is small. Our analysis of the hit position resulted in a probability of hitting a targeted nucleolus of over 70% (certain plus probable hits), confirming the result of one or more DSBs in the nucleolus as shown by the accumulation of γH2AX. This is well in accordance with a prior determination of the probability (more than 80%) of hitting an area of 3 µm with a single ion (Siebenwirth et al., 2015). The main limitations are the accuracy of target position determination and movement of the target between the target definition step and ion delivery. Different DNA densities inside and outside the nucleolus probably account for the slightly reduced measured probability in the present work. When a nucleolus is hit more towards its rim, the γH2AX signal is more likely to be found outside the nucleolus, where more DNA is located and thus marked by γH2AX.

Transportation of chromatin-containing DNA lesions from nucleoli into surrounding perinucleolar regions has previously been observed after I-PpoI treatment (Harding et al., 2015; Van Sluis and McStay, 2015). In our setup, such a process would be expected to decrease the determined probability of a hit with increasing time after irradiation. However, we did not observe such an indication of lesion movement over time. It is possible that apparent lesion migration is linked to nucleoli segregation, but we did not detect this in our experimental system.

Larsen and Stucki (2016) hypothesize that any damage induction in the nucleus outside the nucleoli induces a trans-acting transcriptional response with strongly reduced ribonucleotide incorporation in all nucleoli, even if these were not directly hit. Our observations contradict this hypothesis. None of our cells showed pan-nuclear transcription inhibition, regardless of whether the nucleolus was hit or not. Using doses from 0.3 Gy (1 Cpp) up to 30 Gy (100 Cpp) on the nucleolus, or even 50 Gy (17×10 Cpp) when applied in a cross pattern on the cell nucleus, did not cause a reduction in the 5EU signal (Fig. S3). This leads to the conclusions that even high DSB numbers of up to 3750 DSBs (30 DSBs/Gy×50 Gy×2.5) do not generate enough damage to cause transcription inhibition and that results obtained from laser irradiations might differ from results obtained using ionizing radiation, as already shown in several other examples (Drexler et al., 2015; Kochan et al., 2017). Higher ion numbers were not used in the presented experiment because cells tended to show a pan-nuclear γH2AX signal, making identification of the hit positions impossible (cross irradiations using 10 Cpp are shown in Fig. S3).

The earliest time point we tested for rRNA transcription was 45 min post-irradiation (15 min plus 30 min 5EU incorporation), so it is possible that we missed a transient pan-nucleolar inhibition effect, which has been reported to occur between 30 min and 50 min after γ-irradiation at 5 Gy (Pefani et al., 2018). However, because of the higher doses of up to 50 Gy and the higher relative biological effectiveness of carbon ions (∼2.5), we assume that any damage effects, including the potential inhibition of transcription, occur more prominently and for a longer time period in our setup compared with γ-irradiation experiments. Repair of complex lesions caused by heavy ions is known to occur more slowly than repair of lesions induced by low-LET γ-radiation (Hable et al., 2012). In this regard, we did not observe a significant repair effect in the yield of evaluated foci per irradiated target up to 7 h (Fig. S4D).

Using the ion microbeam, we were able to irradiate a subregion within individual nucleoli. This enabled us to demonstrate that reduced ribonucleotide incorporation does not occur in the whole affected nucleolus, but is restricted to the regions marked by γH2AX. We and others have made similar observations of reduced ribonucleotide incorporation and loss of elongating RNA Pol II at γH2AX-marked chromatin in the cell nucleus outside the nucleoli (Seiler et al., 2011; Penterling et al., 2016; Rona et al., 2018), possibly hinting at similar mechanisms. Further investigations using targeted ion irradiation could help here by inducing localized damage in a certain nuclear region and a certain nucleolus and by allowing simultaneous observation of effects and involved pathways (e.g. ATM and PARP1 dependency).

Formally, we cannot exclude the possibility that radiation-induced damage of proteins or RNA disturbs processes such as pan-nucleolar transcription inhibition or nucleolar segregation, although we consider this unlikely, given the small nucleolar volume affected by traversal of the ions. In addition, from the fact that γH2AX foci formation occurs, we conclude that the initial steps of DNA damage response, such as activation of ATM, function properly and are not inhibited by radiation damage to proteins. Previous work has suggested the involvement of ATM in response to DSB induction in the nucleolus (Kruhlak et al., 2007; Harding et al., 2015; van Sluis and McStay, 2015).

Interestingly, the reduced 5EU incorporation at γH2AX regions after nucleoli irradiation was often accompanied by reduced Parp1 signal. This is apparently in contradiction with the accumulation of Parp1 at damage positions in the cell nucleus seen after laser and ion microirradiation (Mortusewicz and Leonhardt, 2007; Buchfellner et al., 2016). After ion irradiation under conditions similar to those used in the present work, Parp1 foci were detected within seconds of irradiation with doses higher than 300 Cpp and disappeared a few minutes after irradiation (Buchfellner et al., 2016). Release of Parp1 from damage sites is a result of conformational changes in response to substantial auto-poly-ADP-ribosylation (PARylation) (Steffen et al., 2016). As the time of fixation after targeted nucleolus irradiation in the present experiments was at least 45 min, observation of Parp1 accumulation was not expected. Additionally, we assume that the lower dose of 100 Cpp in combination with the high Parp1 background in the nucleolus makes the observation of Parp1 foci unlikely. However, the lack of accumulation does not explain why Parp1 protein is underrepresented at the damage sites at the time points investigated here. Although several interaction partners of Parp1 in the nucleolus have been identified, such as nucleophosmin and TIP5 (Meder et al., 2005; Isabelle et al., 2012), little is known about the subnucleolar localization of Parp1. If Parp1 were mainly associated with chromatin, the continuing presence of PAR chains at the damage site might preclude even a background level of Parp1 binding.

Overall, after ion irradiation of nucleoli we saw reduced transcription locally at DNA damage sites marked by γH2AX, which was accompanied by local Parp1 reduction in about 80% of the cases. In contrast, neither global loss of rRNA transcription activity nor nucleolar segregation could be observed. Therefore, our observations support the assumption that widespread transcription inactivation affecting entire nucleoli is required for nucleolar segregation. A remaining question is why enzyme-induced DSBs elicit these reactions, whereas comparable DSB numbers induced by subnucleolar ion irradiation do not. Responses could depend on the distribution of lesions and portion of the genome affected rather than on total DNA damage levels. Alternatively, enzyme-induced, easily ligatable DSBs and radiation-induced DSBs, which are characterized by unligatable end structures and accompanied by additional lesions, might elicit different response pathways, potentially including use of different DSB repair mechanisms.

## MATERIALS AND METHODS

### Cell lines

For the investigation of potential nucleolar segregation, U2OS cells were used (unmodified cells were a kind gift of Paulius Grigaravicius, Leibniz Institute of Age Research, Jena, Germany). These cells constitutively express a GFP–Mdc1 fusion protein from a CMV promoter (Hable et al., 2012). The fusion protein functions like the endogenous DNA damage marker Mdc1 and is detectable via its fluorescence in live-cell imaging and after fixation.

To investigate the effect of radiation on rRNA transcription, we used stably transfected HeLa-Parp1–CB-tagRFP cells (Buchfellner et al., 2016). A fluorescent, tagRFP-labelled chromobody (ChromoTek) marks the endogenous Parp1 protein in these cells. In this way, nucleoli become visible as targets, as more than 40% of cellular Parp1 is located in the nucleoli (Yung et al., 2004; Rancourt and Satoh, 2009).

HeLa cells were cultivated at 37°C and 5% CO_2_ in DMEM medium (Sigma-Aldrich, D6429). U2OS cells were cultivated in RPMI medium (Sigma-Aldrich, R8758) under the same conditions. FBS at 10% (Sigma-Aldrich, F7524) and 1% penicillin/streptomycin (Sigma-Aldrich, P0781-100ML) were added to both media.

At 12 h before irradiation, 60,000 cells were seeded on the scintillator of a special live-cell imaging (LCI) container (Hable et al., 2009; Siebenwirth et al., 2015), which was pretreated with CellTAK (BD Biosciences, 354240) to enhance cell adhesion. At this point, the media were additionally buffered with HEPES (25 mM; Sigma-Aldrich, H3375) and supplemented with Trolox (0.25 mM; Sigma-Aldrich, 238813).

### Targeted ion irradiation

The irradiation of the cells was conducted with 55 MeV carbon ions at the ion microbeam SNAKE [superconducting nanoscope for applied nuclear (kern-)physics experiments] in Munich, delivering ions in a beam spot size of less than 1 µm full-width at half maximum (FWHM), after targeting several ions to one spot. The targeted irradiation mode was used, which enables hitting regions of 3 µm in diameter, roughly the size of big nucleoli, with more than 80% probability (Siebenwirth et al., 2015). The main causes of deviations were the accuracy of target position determination and target movement in the time period between target definition steps and ion delivery. The LCI container enabled ion detection and high-resolution microscopy with an epifluorescence microscope (Zeiss Axiovert 200 M) at the irradiation setup. According to stopping and range of ions in matter (SRIM) calculations (Ziegler, 2013, www.SRIM.org), after passing the exit foil of 7.5 µm, LCI container foil of 4.7 µm and about 25 µm of medium, carbon ions with an initial energy of 55 MeV have an energy of 43 MeV and LET of about 360 keV/µm in direct proximity to the cell layer. In analogy with the calculations of Hauptner et al., 2006, for single 43 MeV carbon ions, 50% of the dose was concentrated in less than 10 nm radius around the ion trajectory and nearly all ionizations were within a radius of 500 nm. During irradiation, cells were covered with medium at 37°C.

For nucleolus identification, cells were imaged using a 63× oil immersion objective (LCI Plan Neofluar 63×/1.3 M27) in combination with a 0.63× camera adapter and a 20HE filter set and 555 nm LED (all Zeiss). Thus, the field of view of the camera (AxioCam Mrm3 Rev3, Zeiss) was 225×170 µm^2^. Single slice images were taken, because minimizing the time for target identification was more important than improvement of image quality by deconvolution of 3D stacks.

Composed fields consisting of 2×2 microscope camera images were irradiated. In the single field of view of the camera, the biggest nucleolus in each cell nucleus was irradiated with a defined ion number on one spot. Nucleoli were identified by the fluorescence signal of Mdc1-GFP, appearing as dark spots in the nucleus, or by the Parp1–CB-tagRFP signal, appearing as bright spots in the nucleus. If it was not possible to identify the nucleoli (e.g. because of different expression levels of the fluorescent protein), the cell nucleus was irradiated with a cross pattern consisting of 17 spots. Thus, these nuclei were clearly marked and analysed separately. In successive irradiation experiments, the number of applied carbon ions per targeted spot was 1, 10, 50 and 100 carbon ions per target spot. This roughly equalled 0.3–30 Gy on the nucleus.

The irradiation of a composed field containing about 70 cells lasted approximately 10 min. The composed fields were irradiated one after another in defined time steps while remaining at 37°C in the irradiation setup. This scheme had the advantage that cells treated at different doses and time points partly undergo the same detection procedure. This allowed irradiation of more cells, in contrast to fixing each single time point on one sample. For recovery times longer than 4 h, samples were removed from the irradiation setup and incubated at 37°C and 5% CO_2_ after the irradiation. In total, two samples covered the recovery times from 15 min to 3 h 15 min, one sample the period from 6–7 h and two samples the period of about 24 h.

### UBF protein staining and 5EU transcription assay

For investigation of UBF redistribution after targeted irradiation of the nucleolus, the medium of the cells was changed after irradiation and cells incubated for 2 h at 37°C and 5% CO_2_. Cells were fixed for 15 min with 2% PFA at room temperature. Cells were washed once with PBS and subsequently washed three times for 5 min with PBS containing 0.15% Triton X-100. Then cells were then washed three times for 10 min with PBS^+^ (PBS containing 1% BSA and 0.15% glycine) to block unspecific binding. Subsequently, cells were incubated overnight at 4°C with 100 µl of a primary antibody mix against UBF (1:200, mouse, F-9; Santa Cruz Biotechnology, catalogue no. sc-13125) and γH2AX (1:200, rabbit; Abcam 81299) in PBS^+^. The following day, cells were washed once for 5 min with PBS, once for 10 min with PBS containing 0.15% Triton X-100, once again for 5 min with PBS and then blocked for 7 min with PBS^+^. Secondary antibody conjugates Alexa Fluor 647 (goat-anti-mouse; Molecular Probes #A21237) and Alexa Fluor 488 (goat-anti-rabbit; Molecular Probes A11034) were diluted 1:200 in PBS^+^. The samples were incubated with 100 µl of the antibody solution for at least 45 min at room temperature. After the final washing steps of 2×10 min with PBS containing 0.15% Triton X-100, followed by 1×10 min and 2×7 min with PBS, nucleoli were counterstained by incubation with 300 µl Syto83 (1:5000; Thermo Fisher Scientific, catalogue no. S11364) in PBS for 20 min. The whole procedure was performed in the LCI container mentioned above. Finally, the liquid was removed and the sample was mounted with ProLong Gold (Thermo Fisher Scientific) mounting medium and secured with a cover glass. Control experiments after 2 h of actinomycin D treatment (30 nM) were performed with the same protocol and using the following antibodies kindly provided by Brian McStay (Centre for Chromosome Biology, National University of Ireland Galway, Galway, Ireland): anti-UBF (sheep, 1:200), anti-Nop52 (sheep, 1:500), anti-fibrillarin (mouse, 1:200); anti-sheep-Alexa Fluor 647 (donkey, 1:200) and anti-mouse-Alexa Fluor 488 (donkey, 1:200). Primary antibodies introduced in Van Sluis and McStay (2015). Secondary antibodies are also commercially available from Jackson ImmunoResearch (anti-sheep-Alexa Fluor 647, 713-605-003; anti-mouse-Alexa Fluor 488, 715-545-150).

To quantify the rRNA transcription, the Click-iT RNA Alexa Fluor 488 Imaging Kit (Thermo Fisher Scientific, C10329) was used. This allowed measurement of incorporation of the nucleoside analogue 5EU into the RNA. After post-irradiation incubation of the sample for varying periods of time, the cells were pulse-labelled for 30 min with 1 mM 5EU. After fixation of the sample for 15 min with 2% PFA, IF staining of γH2AX was conducted as described above utilizing anti-γH2AX (1:200, mouse; Abcam 81299) and Alexa Fluor 647 (1:200, goat-anti-mouse; Molecular Probes A21237,). After IF staining, click chemistry staining of 5EU was done using the Click-iT kit according to the manufacturer's instructions. All liquid was removed and the sample incubated for 15 min in PBS containing 0.15% Triton X-100. Alexa Fluor 488 Click-iT reaction cocktail (500 µl) was prepared as suggested by the kit manual. Subsequently, the sample was washed with PBS and cells incubated in the reaction cocktail for 30 min at room temperature while protected from light. Finally, the sample was washed with Click-iT reaction buffer and mounted on a cover slip with ProLong Gold.

### Microscopy

Protein distribution and rRNA transcription were visualized by epifluorescence microscopy using a Zeiss AxioObserver Z.1 microscope, using a 63× objective (63×/1.4 NA Plan-Apochromat; oil) equipped with a Colibri LED light source. A 20HE filter set was used for the tagRFP signal of Parp1 or Syto83, filter set 13 for Alexa Fluor 488 (5EU) and filter set 50 for Alexa Fluor 647 (γH2AX) (all Zeiss). The used filter sets exclude crosstalk between the tagRFP and the Alexa Fluor 488 channel. For rRNA transcription, nucleoli were verified by phase contrast imaging. The whole cell height was imaged by *z*-stack images with 300 nm offset. The stacks were deconvolved with Huygens deconvolution software (Scientific Volume Imaging) to improve depth resolution. The irradiation fields were reconstructed by using specific cross marks on the scintillator. Cell images after IF staining were individually registered with the Parp1 live-cell images taken before irradiation. Criteria for reconstruction were cell position, shape and number of nucleoli, and their corresponding sizes. About one-third of the irradiated cells could not be reconstructed reliably and were excluded from analysis. This included all cells with recovery times of 24 h, because of cell movements and loss of γH2AX signals after successful DSB repair.

For image analysis, the free software ImageJ (https://imagej.nih.gov/ij/) was used.

## Supplementary Material

Supplementary information
