# Supplementary material for: Local inhibition of rRNA transcription without nucleolar segregation after targeted ion irradiation of the nucleolus
Source: J Cell Sci. 2019 Oct 9;132(19):jcs232181. doi: 10.1242/jcs.232181 (PMC6803363; doi:10.1242/jcs.232181)
Supplement: Supplementary information [file joces-132-232181-s1.pdf]

SUPPLEMENTARY DATA

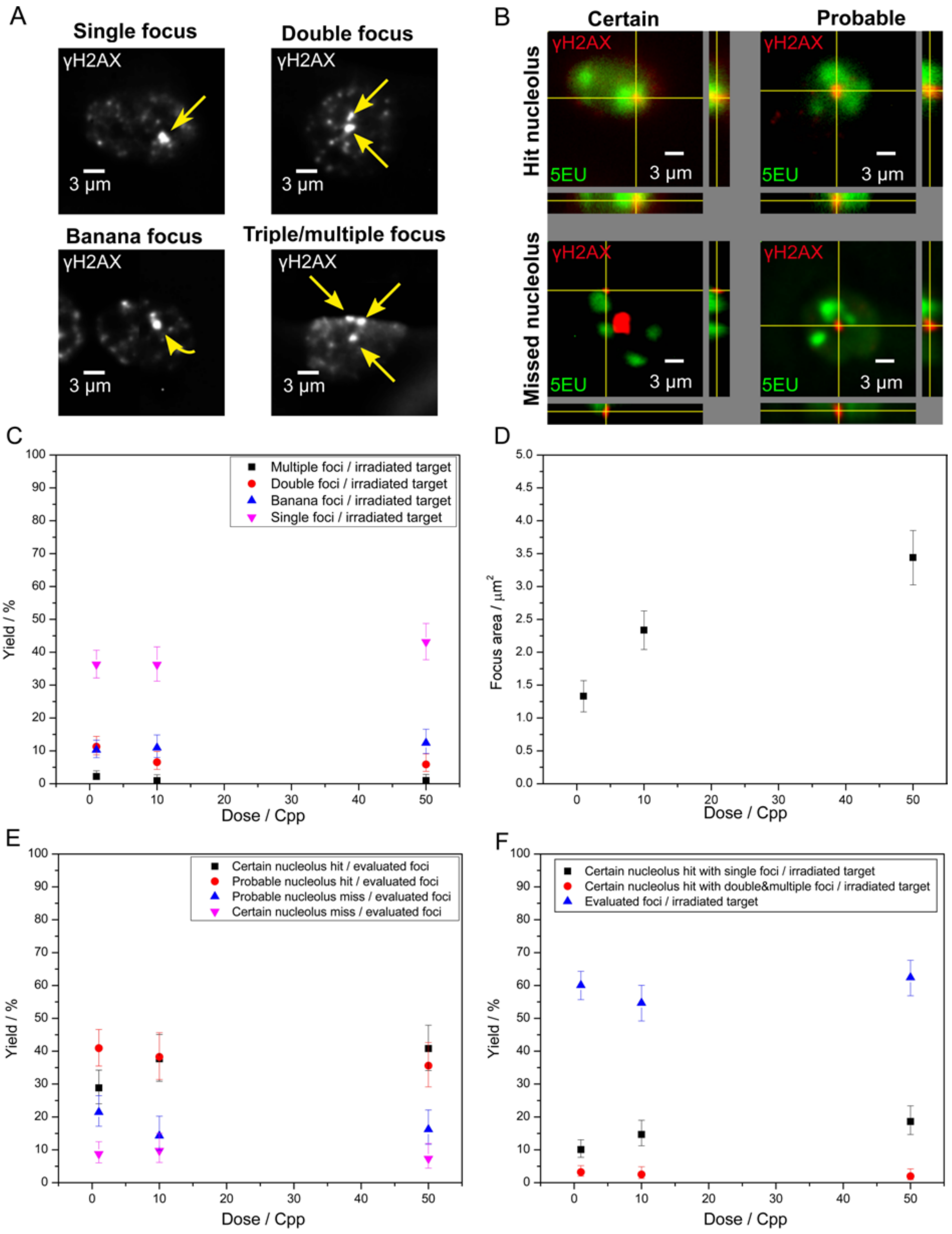

Fig. S1: Analysis of targeted nucleolus irradiation with 1, 10 and 50 carbon ions per point (Cpp)

Journal of Cell Science • Supplementary information

A: Foci categories in 5EU transcription analysis using γH2AX signal after irradiation of 1, 10, 50 or 100 carbon ions on a single spot (Cpp) of a nucleolus.

B: Four hit categories by relating the 5EU nucleolus signal with the γH2AX damage signal after irradiation of 1, 10, 50 or 100 carbon ions on a nucleolus (Cpp) (Certainly/probably hit nucleolus and certainly/probably missed nucleolus). The example of the certainly missed nucleolus, shows two γH2AX foci, which originate from a 1 and 10 Cpp irradiation and locate next to their targeted nucleoli. The cell height seems to shrink during IF staining from 6-7 µm during live cell imaging to about 3 µm, which was verified by phase contrast images. Thus, analysis of DNA damage above and below nucleoli was not possible. Z-stacks shown consist of 13 slices with distance 300 nm, containing of the single nucleus. Scale bars are 3 µm and are valid for all cross sections.

C: Frequency of single, double, banana and triple/multiple foci.

D: Average focus area of single foci with certain nucleolus hit. Error bars describe the standard error of the mean at 95% confidence.

E: Frequency of certain/probable nucleolus hits and certainly/probably missed nucleoli.

F: Frequency of nucleolus hits with single or multiple foci per irradiated target. Additionally, the ratio of evaluable foci per irradiated target independent from the hit position is shown. Since only few cells were irradiated with 100 Cpp, in these analyses evaluation was restricted to cells treated with doses up to 50 Cpp.

Error bars of C, D and F describe the 95% confidence Wilson score intervals of the binomial proportion.

Journal of Cell Science • Supplementary information

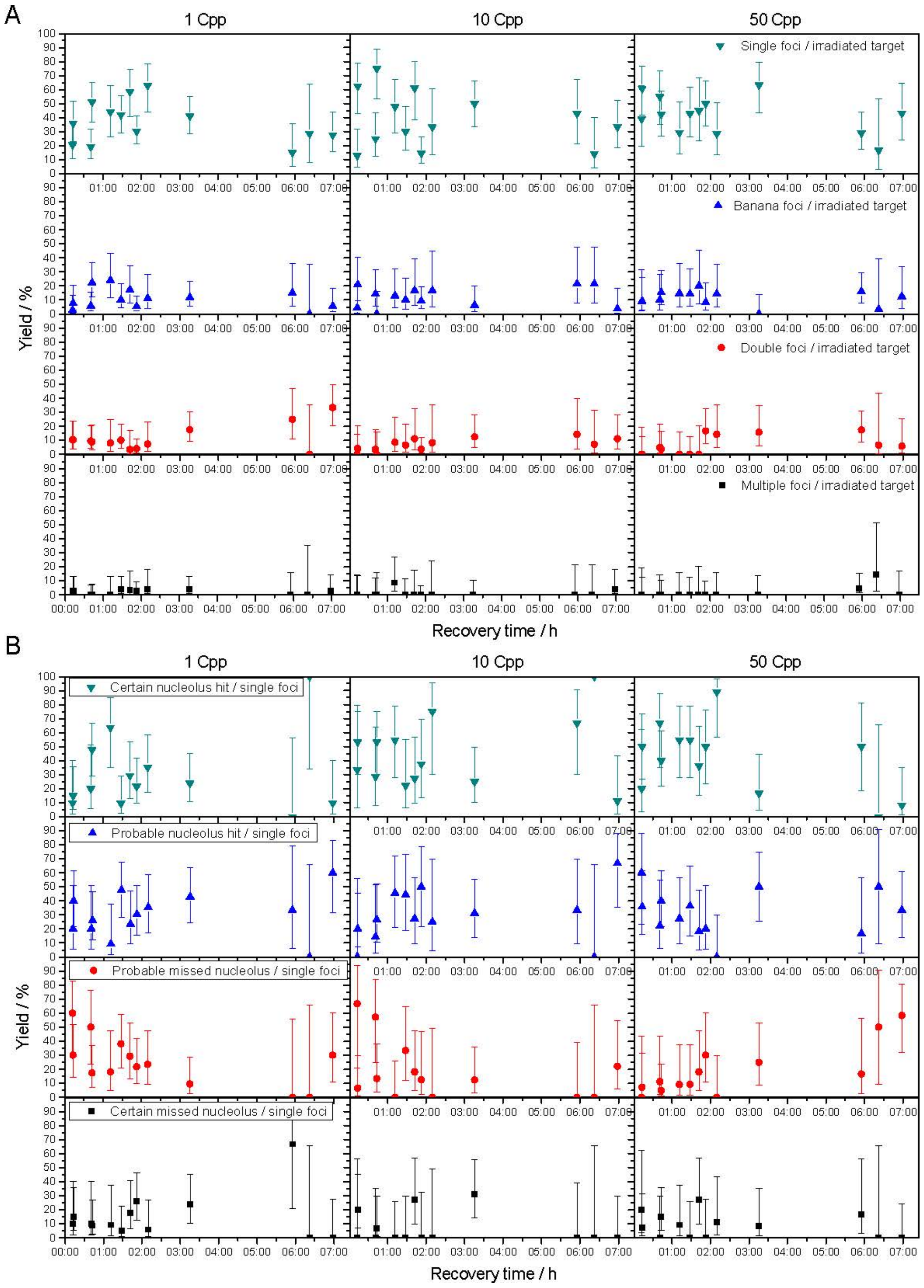

Fig. S2:

A: Frequency of single, banana, double and multiple foci regarding time and dose (left to right column). Error bars describe the 95% confidence Wilson score intervals of the binomial proportion. Because the yields seem to be independent from time within the range of uncertainties, data of time

Journal of Cell Science • Supplementary information

points were pooled to investigate dose effect (Fig. S1 D). Since only few cells were irradiated with 100 Cpp, in these analyses evaluation was restricted to cells treated with doses up to 50 Cpp.

B: Frequency of single foci with certain/probable nucleolus hits and certainly/probably missed nucleoli regarding time and dose (left to right column). Error bars describe the 95% confidence Wilson score intervals of the binomial proportion. Because no significant difference in time was observed, data of time points were pooled to investigate dose effect (Fig. S1 F). Since only few cells were irradiated with 100 Cpp, in these analyses evaluation was restricted to cells treated with doses up to 50 Cpp.

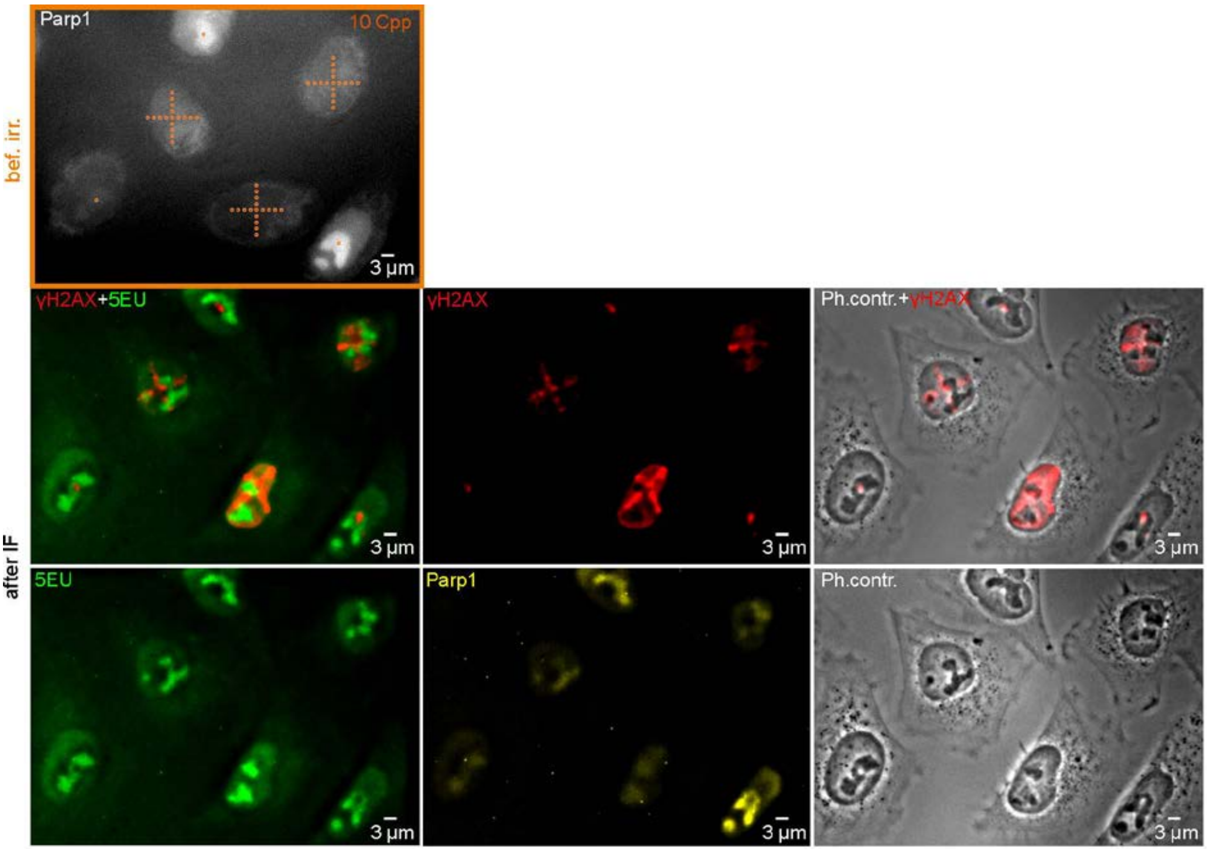

Fig. S3:

Targeted irradiation of nucleoli in HeLa-parp1-CB-tagRFP with 10 Cpp and 2 h recovery time. For target definition the live cell image of Parp1 was used (orange). 2 h after irradiation cells were incubated with 5EU for 30 min, fixed and IF staining of γH2AX performed. Irradiation of cross patters consisting of 17 points allowed to check for an overall effect on the rRNA transcription after introducing DNA damage in the nucleus. Neither targeted nucleoli irradiation nor cross pattern irradiation showed an impact on 5EU incorporation. At the γH2AX damage sites of the irradiated nucleoli, however, the 5EU and Parp1 signal is significantly reduced. This is accompanied by brighter sites in the phase contrast image.

Journal of Cell Science • Supplementary information

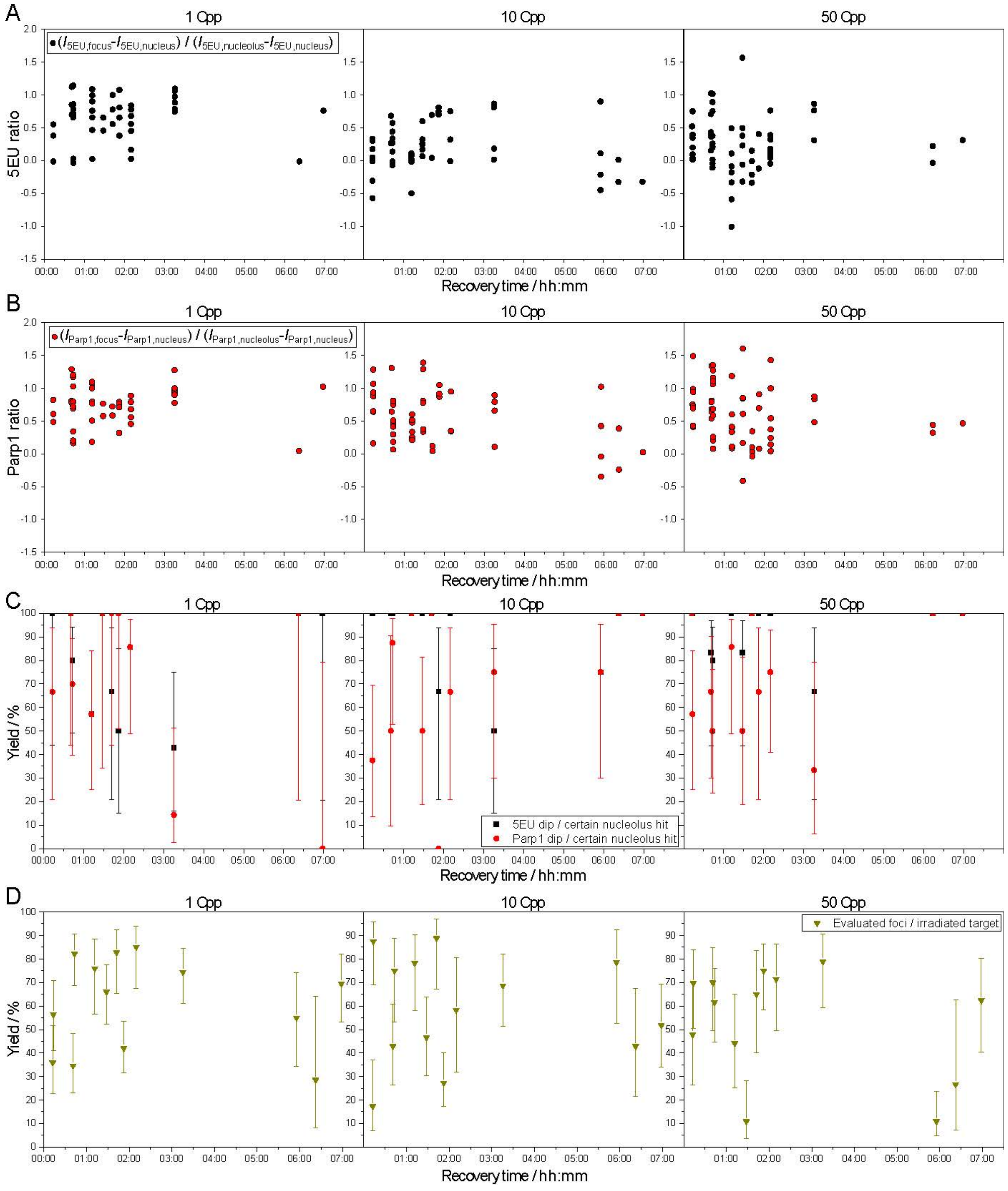

Fig. S4:

A&B: 5EU (black) and Parp1 intensity (red) (I_{5EU}, I_{Parp1}) ratios at single foci marking a certain nucleolus hit regarding dose and time. Calculated was the ratio of the signal intensity at the focus $I_{...,focus}$ subtracted by the nucleus background signal $I_{...,nucleus}$ compared to the signal intensity in the residual nucleolus $I_{...,nucleolus}$ subtracted by the nucleus background signal $I_{...,nucleus}$. 5EU and Parp1 signal are significantly reduced at the DNA damage site marked by γH2AX. For higher rates of 10 and 50 Cpp 5EU incorporation significantly decreases to about 20%. Error bars describe the standard error of the mean at 95% confidence. Within the range of variances, the data is independent of time and thus, it was pooled to investigate a dose effect (Fig. 3 B).

C: Frequency of 5EU/Parp1 dips per certain nucleolus hit regarding time and dose (left to right column). Error bars describe the 95% confidence Wilson score intervals of the binomial proportion. Within the range of uncertainties, data is independent of time and thus, it was pooled to investigate a dose effect (Fig. 4).

D: Frequency of evaluated foci per irradiated target regarding time and dose (left to right column). Error bars describe the 95% confidence Wilson score intervals of the binomial proportion. Because no significant difference in time was observed, data of time points were pooled to investigate dose effect (Fig. 3 F). Since only few cells were irradiated with 100 Cpp, in these analyses evaluation was restricted to cells treated with doses up to 50 Cpp.

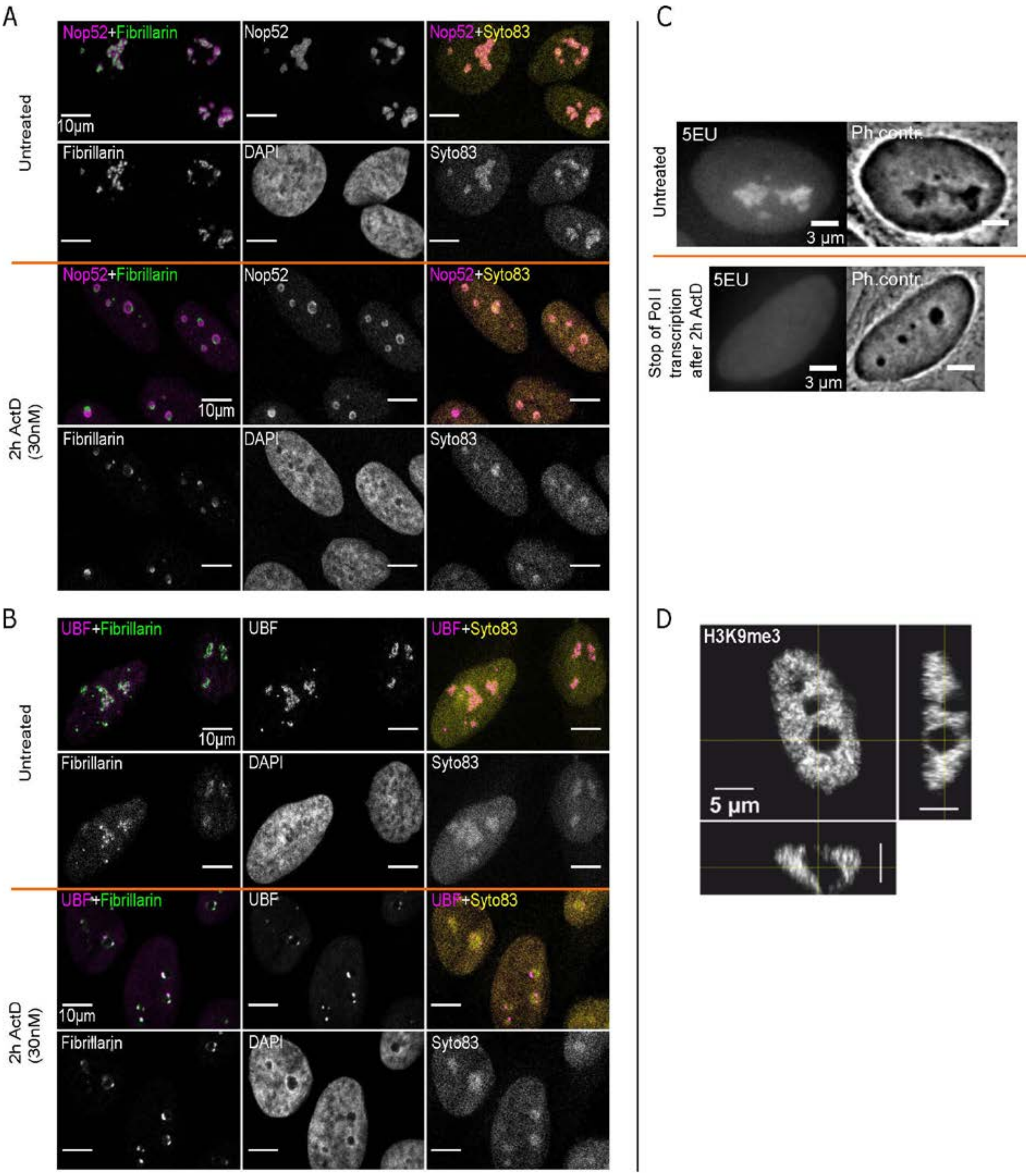

Fig. S5:

A&B: Nucleolar segregation imaged at a confocal microscope (LSM 700, Zeiss GmbH). Image rows above orange line show normal Nop52/UBF (A/B) and Fibrillarin distribution in the nucleoli. Image rows beyond orange line show reorganisation of UBF and Fibrillarin into caps at the periphery of the nucleoli after inhibition of rRNA transcription with 2h Actinomycin D (30 nM), In parallel, Nop52 and Syto83 still mark the central area of the nucleoli.

C: Visualization of Pol I rRNA transcription by 5EU assay. Left image normal 5EU signal in the nucleoli. Right image shows loss of the 5EU Signal after 2h Actinomycin D treatment (30nM). Nucleoli were still observable in phase contrast image, but show a more spherical shape than untreated.

Journal of Cell Science • Supplementary information

D: Z-stack of a HeLa cell with stained histone methylation H3K9me3 imaged at a confocal microscope. Nucleoli are visible as dark spheres in the cell nucleus with about 50% of the height of the cell nucleus.

Journal of Cell Science • Supplementary information
